# Effectiveness of a multi-component sleep-mood group intervention on improving insomnia in university students – a pilot randomized controlled trial

**DOI:** 10.1186/s40359-024-02057-1

**Published:** 2024-11-05

**Authors:** Laura M. Pape, Sophie Jonker, Liia M.M. Kivelä, Annemieke van Straten, Niki Antypa

**Affiliations:** 1 Department of Clinical Psychology, Leiden University, Leiden, The Netherlands; 2 Department of Clinical, Neuro- and Developmental Psychology, VU Amsterdam, Amsterdam, The Netherlands

**Keywords:** Insomnia, Mood, Mindfulness, University students, Group intervention

## Abstract

**Background:**

Sleep and mental health problems are very common in university students. The objective of this study was to assess the effectiveness of a multi-component sleep-mood intervention on improving sleep and mental health in university students with clinically significant insomnia symptoms, and to investigate possible mediators.

**Methods:**

Thirty-five participants were randomized to the Sleep Mood Intervention: Live Effectively (SMILE) intervention (*n* = 23), or wait-list group (*n* = 12). SMILE is a multi-component group therapy and includes elements of cognitive behavioral therapy for insomnia (CBT-I), mindfulness, and lifestyle modifications, in four weekly two-hour sessions. The primary outcome was insomnia severity. Secondary outcomes were severity of depression and anxiety, and quality of life (QoL). Dysfunctional beliefs and attitudes about sleep and pre-sleep arousal were assessed as mediators.

**Results:**

Intention-to-treat analysis showed significant time x treatment interaction on insomnia symptoms *p* = .02, *partial η²*= 0.15, *d* = 0.84 [95% CI: 0.63 to 1.14], with significantly lower insomnia severity for SMILE compared to waitlist at post-test. No significant effects were found on depression *d* = 0.02 [95% CI: -0.35 to 0.37], anxiety *d* = 0.15 [95% CI: -0.16 to 0.53], and QoL *d* = 0.09 [95% CI: -0.25 to 0.42]. Dysfunctional beliefs mediated the effect on insomnia severity, but pre-sleep arousal did not.

**Conclusions:**

This integrated group intervention is associated with reductions in insomnia symptoms in university students. Since no significant effects were detected on mood and QoL, future studies with larger sample size may explore the effects of this intervention on these outcomes.

**Trial registration:**

Registry: Overzicht van Medisch-wetenschappelijk Onderzoek. Registration number: NL-OMON46359. Date of registration: September 18^th^, 2018.

## Background

Insomnia is a major public health problem and can, if untreated, lead to a range of physical and mental health problems, such as hypertension, obesity, cardiovascular disease, depression and anxiety disorders [1–3]. Sleep problems are especially common among university students [4, 5]. A systematic review of the prevalence of insomnia in 16,748 university students found a weighted mean of 18.5% (ranging from 9.4 to 38.2%), which was substantially higher than in the general population at 7.4% [6].

The increased prevalence of insomnia may be attributed to numerous challenges in the lives of students. University students face high expectations of their education and future careers, have to develop greater personal independence, and face the challenge of balancing social activities, part-time jobs, and self-directed studying, all while contending with financial pressure due to rising tuition fees and living expenses [7]. These generally conflicting demands can lead to excess stress among students, making them a high-risk population for both sleep disturbances and mental health complaints [8]. Moreover, university students are in an age group that is particularly vulnerable to the onset of psychopathology [9].

A growing body of literature supports the effectiveness of psychological interventions for insomnia, especially cognitive behavioral therapy for insomnia (CBT-I) [10–12]. In CBT-I, patients learn to identify and modify maladaptive thoughts, attitudes, and behaviors, by utilizing psychoeducation, cognitive restructuring, stimulus control, sleep restriction, and relaxation techniques. It has been shown effective in both individual and group face-to-face formats as well as in online formats [10, 13, 14], and even for those with subthreshold insomnia [15]. However, although most people with insomnia benefit from CBT-I only a small proportion (15–30%) achieves full remission as discussed in a prior review [16]. Furthermore, some groups benefit less than others. Meta-analyses on sleep interventions including CBT-I in student populations show smaller effect sizes compared to the general adult population [10, 13, 17]. The meta-analysis by Chandler et al. almost exclusively included CBT-I studies and found a moderate effect size (*d* = 0.55), while the meta-analyses of Saruhanjan et al. and Kodsi et al. included more studies with different types of sleep interventions, such as sleep education and relaxation training, and found moderate effects on sleep, with effect estimates of 0.61 and 0.51, respectively. This is consistently lower than the effects seen in the general population, for instance, a meta-analysis including 87 studies by van Straten et al. showed a large effect size (*g* = 0.98). A possible explanation for the smaller effect size in students may be that several interrelated factors are especially relevant in the lives of students that are insufficiently addressed in current interventions. For instance, issues relevant to students include perfectionism, stress-sleep reactivity, poor psychological flexibility, and coping skills, which are known to contribute to the development and maintenance of sleep problems [5, 18–20] and may have to be emphasized more in a student population. Students may require a tailored approach that considers their specific challenges and circumstances by using cognitive-behavioral techniques to target not only sleep but also associated problems such as perfectionism and maladaptive coping. Interventions targeting multiple behaviors and skills simultaneously may therefore be more effective than single-component treatments to promote better sleep among students.

A promising add-on to CBT-I is mindfulness-based interventions. Mindfulness is a practice aiming to direct an individual’s attention to the present moment without judgment and increase awareness of internal and external experiences [21]. It is used to decrease stress and increase mental well-being through exercises such as breath regulation and body-scan meditation [22]. A recent meta-analysis of seven RCT’s showed that mindfulness-based stress reduction significantly improved sleep quality, depression, and anxiety among adult insomnia patients [23]. Another study among adolescent girls examined the effectiveness of a multi-component mindfulness-based group intervention and found that the intervention was feasible and resulted in moderate improvements in subjective sleep [24]. Therefore, in order to tackle the stress-related factors mentioned above that are intertwined with the lives of students, mindfulness-based techniques might be a fruitful add-on to sleep interventions.

A brief group multi-component sleep-mood intervention combining CBT-I and mindfulness practices with lifestyle components, the Sleep Mood Intervention: Live Effectively (SMILE) intervention, was developed to target sleep and mood in university students, taking into account the needs of this target group. The objective of the present study was to assess the effectiveness of the SMILE intervention in university students with sleep complaints. The primary outcome was insomnia severity. The secondary outcomes were symptoms of depression, anxiety and quality of life. Given the close relationship between sleep and mental health, we hypothesized that SMILE improves all outcomes. Secondly, we explored the mechanisms of change, namely whether the treatment effect on insomnia was mediated by dysfunctional beliefs about sleep and levels of pre-sleep arousal. Both are factors that have been previously assessed as mediators in other intervention studies on sleep outcomes [25].

## Methods

### Study design

The current research is a pilot randomized controlled trial to determine the effectiveness of a multi-component sleep-mood intervention (the SMILE intervention; Sleep Mood Intervention: Live Effectively) in university students on reduction of insomnia symptoms comparing two groups: the SMILE intervention group and a waiting list control group with an allocation ratio of 2:1. This ratio was used for clinical purposes, in order to provide help to more students. The study included a baseline period of one week, an intervention period of four weeks, and a post-intervention period of one week. Participants in the wait-list condition received the SMILE intervention after six weeks. The study was approved by the Medical Ethical Committee Leiden The Hague Delft (METC-LDD) in the Netherlands (NL64330.058.17, September 18^th^, 2018) and was registered at the Overzicht van Medisch-wetenschappelijk Onderzoek register (registration number: NL-OMON46359) on September 18^th^, 2018. This study was carried out in accordance with the Declaration of Helsinki and the guidelines of Good Clinical Practice (GCP).

### Recruitment and participant screening

Participants were recruited between September 19^th^, 2018 and February 11^th^, 2020. Recruitment took place at Leiden University through posters placed in university buildings, on social media via postings in student groups, as well as through referrals from other studies. The recruitment process consisted of two stages. First, pre-screening of participants was performed through online questionnaires, including the Insomnia Severity Index (ISI) with a cut-off of ≥ 10, to identify students with current sleep problems. Second, all participants deemed eligible through the pre-screening procedure and willing to participate in the study completed a face-to-face diagnostic interview with the M.I.N.I Plus International Neuropsychiatric Interview [26] confirming that the participants met the full inclusion and exclusion criteria. The diagnostic interview was performed by a psychologist or a Master-level psychologist in training (with possibility to consult with the psychologist after the intake).

Inclusion criteria were (1) self-reported sleep complaints with ISI score of ≥ 10, representing clinically significant insomnia [27]; (2) being enrolled as a university student (3) being 18 years or older; (4) adequate proficiency in both written and spoken English; and (5) willingness to participate in a four-week group intervention program and giving informed consent. Students were excluded under the following circumstances: (1) in the presence of clinically significant psychopathology (as based on DSM-IV criteria from the M.I.N.I. diagnostic interview) regarding: current Major Depressive Disorder, Bipolar Disorder, Panic disorder, Social Anxiety Disorder, Post-traumatic Stress Disorder, Attention Deficit Hyperactivity Disorder, Eating disorders, and Psychotic disorders; (2) in the presence of a sleep disorder such as narcolepsy or sleep apnea; (3) in the presence of acute somatic illness that might interfere with the intervention; (4) currently (past month) using medication known to influence sleep (e.g. hypnotics, anxiolytics, antidepressants, stimulants, and > 0.5 mg melatonin per day), except for antidepressant treatment which if it was started more than 3 months prior to study enrollment and dosage was stable then the participant was included; (5) current substance use dependency; and (6) concurrent psychotherapy (e.g., CBT, including past CBT for sleep or depression).

**Fig. 1:**
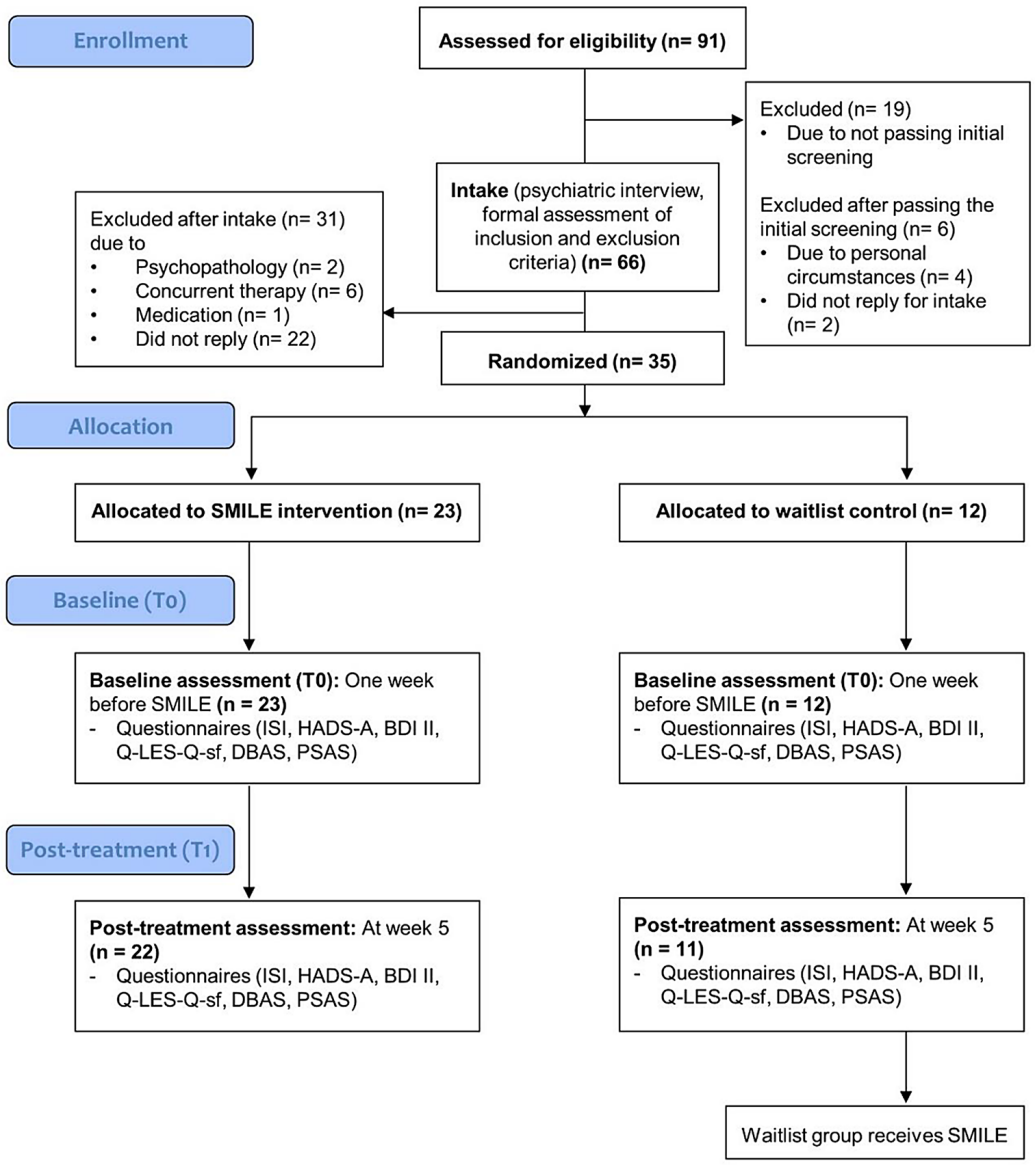
Overview of the study design - CONSORT flow diagram. *Note*. Flow diagram of participants in the study. ISI = Insomnia Severity Index; HADS-A = Hospital Anxiety and Depression Scale – Subscale Anxiety; BDI II = Beck Depression Inventory II; Q-LES-Q-sf = Quality of Life Enjoyment and Satisfaction Questionnaire; DBAS = Dysfunctional Beliefs and Attitudes about Sleep Scale; PSAS = Pre-Sleep Arousal Scale

An overview of the study design can be found in Fig. 1. The online eligibility screening was completed by 91 students, of which 19 were excluded due to scoring < 10 on the ISI, and six due to other reasons (Fig. 1). Sixty-six students were invited to the intake and completed the psychiatric interview. After this intake, 31 students were excluded due to exclusion criteria during or after the initial screening. Thirty-five people were randomized into SMILE (*n* = 23) or waitlist group (*n* = 12). We used block randomization with 24 sets of numbers and 3 numbers per set ranging from 1 to 3 in random order, with 1 = intervention, 2 = intervention, 3 = waiting list. An independent researcher who had no involvement in the current research did the random allocation sequence which was computer-generated using *randomizer.org*. Allocation concealment was ensured since the independent researcher was in complete charge of the randomization process and its sequences, and the researchers of the study had no access to this. Research assistants enrolled participants in the study and sent the participant numbers to the independent researcher who assigned them to the conditions. Groups could start when enough participants had been enrolled to form groups, this took on average 4–6 weeks to achieve. Of the 11 participants remaining in the waiting list group 10 proceeded with the intervention.

### Intervention

The SMILE intervention (Sleep Mood Intervention: Live Effectively) is a group therapy intervention with a duration of four weeks (two-hour sessions weekly). Each session covered different topics: Session (1) Sleep education, sleep hygiene, stimulus control, and sleep restriction; Session (2) negative thoughts; Session (3) relaxation techniques and mindfulness; Session (4) lifestyle issues. Further information on the content of the SMILE intervention can be found in Table 1. The intervention sessions took place at the Leids Universitair Behandel- en Expertise Centrum (LUBEC) and the last session online due to the COVID pandemic. Each group had a minimum of four and maximum of six participants. The groups were led by therapists who were trained Master-level psychologists. They were instructed to follow the intervention protocol. Themes were addressed in the form of an oral presentation by the therapist and group discussions. Generally, participants were actively involved in the sessions. Practical exercises were included in each session, either individually (e.g., with worksheets, self-reflection) or carried out collectively in the group setting (e.g., breathing exercises, group discussions). Sessions started with a greeting of the group and an evaluation of the past week, followed by an overview of the aims of the session (see Table 1), practical exercises, group discussion, and the concluding discussion covering key takeaways, homework assignment, and preparations for the next session. In the third session, mindfulness and breathing exercises were carried out for the whole group upon the guidance of the therapist. In the fourth session (lifestyle issues), a range of student-relevant issues were covered (see Table 1 for overview, topics were chosen according to the needs of the specific group). Each student created a personalized relapse prevention plan in collaboration with the therapist. Brief 1:1 substitute sessions were offered in case a participant could not attend the group session reviewing the relevant materials.

**Table 1:** Content of the SMILE intervention

Session	Content
Session 1	**Introduction – Sleep education and hygiene** - Biology of sleep, effects of sleep deprivation, importance of sleep in physical and mental wellbeing - Sleep hygiene (behaviours that promote healthy sleep), e.g., sleep schedule, avoiding daytime napping, and limiting coffee and substance use - Stimulus control (strengthen the bed as a cue for sleep) - Sleep restriction (limiting the hours in bed) - *Homework*: Applying the sleep hygiene rules and adjusting the sleep schedule based on sleep restriction if necessary
Session 2	**The Mind – Dealing with negative thoughts** - Concept of cognitive arousal and its impact on sleep - Cognitive behavioural techniques (e.g., thought records) - Dysfunctional thoughts about sleep or non-sleep related - Constructive worrying and positive refocusing - *Homework*: To complete the thought record exercise and/or the constructive worrying exercise
Session 3	**The Body – Relaxation and Mindfulness** - Dealing with stress and arousal - Deep (diaphragmatic) breathing - Progressive muscle relaxation - Mindfulness practice directed at challenging automatic reactions to stress, increasing awareness to the present moment, and letting go of negative thoughts associated with sleep - *Homework*: Practicing a mindfulness or relaxation exercise at least once a day through one of the suggested exercises. Filling out the mindfulness worksheet and the relaxation worksheet
Session 4	**The Whole – Lifestyle Issues** - Cognitive and behavioural patterns influencing sleep - Dysfunctional coping, such as substance use (alcohol, drugs, smoking) and emotional eating - Lifestyle topics such as flexibility and planning, perfectionism, burnout, diet, self-acceptance - Emotion focusing and processing - Summary and relapse prevention plan - *Homework*: Applying the knowledge and exercises of this intervention in daily life and using the relapse prevention plan

### Measurements

#### Assessment points

Assessments took place at baseline (T0) and after five weeks (T1). After T1, all participants in the waitlist control group were offered the SMILE intervention, which finished the controlled element of the trial. Demographic characteristics were assessed at baseline. Questionnaires were assessed using the cloud-based platform Qualtrics.

#### Primary outcome

Insomnia complaints were measured with the 7-item ISI [27]. A higher score suggests more insomnia severity in the past week or past two weeks, with total scores ranging from 0 (no insomnia) to 28 (severe insomnia). A cut-off score of 10 was determined as optimal to indicate clinical levels of insomnia and was therefore used as a cut-off for inclusion of participants [27]. The ISI is a widely used measure with adequate internal consistency and reliability [28]. The internal consistency in this sample at baseline was moderate at Cronbach’s α = 0.65.

#### Secondary outcomes

The Beck Depression Inventory II is a 21-item questionnaire which measures depressive symptoms in the preceding two weeks. Total scores are ranging from 0 (no depression) to 63 (severe depression). This inventory is shown to be a sensitive and reliable measure [29]. The internal consistency in this sample was good at Cronbach’s α = 0.84.

Anxiety symptoms were assessed using the anxiety subscale of the Hospital Anxiety and Depression Scale (HADS-A) [30]. Seven items are assessed to measure anxiety over the past week. The total score ranges from 0 (no symptoms of anxiety) to 21 (severe symptoms of anxiety). Internal consistency of the HADS-A in this sample was moderate, with Cronbach’s α = 0.76.

Quality of life was measured with the Quality of Life Enjoyment and Satisfaction Questionnaire - short form (Q-LES-Q-sf) [31]. The Q-LES-Q contains 16 item-domains, including physical health, mood, work, social relations, ability to function in daily life and more. Totals scores range from 14 to 70, with higher scores indicating better quality of life. The internal consistency in our sample was satisfactory at α = 0.78.

#### Other outcomes

Dysfunctional Beliefs and Attitudes of Sleep (DBAS-16) were assessed as a mediator variable. The DBAS consists of 16 items evaluating beliefs, expectations, and attitudes about sleep complaints [32]. Each item statement is rated on a scale from 0 (strongly disagree) to 10 (strongly agree), and higher scores indicate more dysfunctional beliefs and attitudes. The questionnaire is found to have adequate psychometric properties [32]. The internal consistency in our sample was satisfactory at Cronbach’s α = 0.77.

Sleep-related arousal was measured with the Pre-Sleep Arousal Scale (PSAS), which measures subjective arousal before sleep in 16 items [33]. Each item is rated on a scale from 1 (not at all) to 5 (extremely).We reported the sum score of the PSAS (ranging from 16 to 80). Higher scores indicate more pre-sleep arousal. The internal consistency was good with Cronbach’s α = 0.87.

### Deviations from the study protocol

A total sample size of 72 participants was required to have sufficient power (0.80) to be able to detect differences of a small effect size (*d* = 0.17) using alpha < 0.05 and repeated measures ANOVA. This was calculated using the G*Power v3.1.9.2. tool. However, there was a premature termination of the study due to the COVID-19 pandemic. Organization of the group session in an online format was not possible, not only due to a lack of resources, but also as it would have resulted in fundamental changes to the intervention content. After the COVID-19 break out (March 2020), the recruitment stopped. Furthermore, in the study protocol, subjective sleep quality assessed with a sleep diary was listed as another primary outcome measure. However, due to large amounts of missing data (14 participants (40%) had sufficient data at both pre-test and post-test) data imputation was not possible and therefore this outcome was not analyzed and not reported.

### Statistical analysis

All analyses were performed as intention to treat in IBM SPSS Statistics (version 27.0). Analyses were carried out using a 0.05 α-level (two-tailed). Baseline differences between the two groups were tested with independent samples, t-tests for continuous variables, and chi-square tests for categorical variables. Data was checked for outliers and for assumptions for parametric analyses. Last observation carried forward was used to impute missing values at post-test (*n* = 2).

The primary and secondary outcomes were analyzed using a repeated measures analysis of variance (ANOVA), with condition (SMILE versus waitlist) as between-subjects factor, and time (T0 versus T1) as within-subjects factor. The magnitude of the effect for the within-group change and difference between groups at post-test was calculated using Cohen’s d, with 0.2, 0.5 and 0.8 as small, moderate and large effects, respectively [34]. Participants were categorized as a remitter when the ISI score at post-test was below 10 points, and participants were categorized as treatment responders when there was a reduction in the total ISI score of ≥ 8 points [27].

For mediation analysis of DBAS and PSAS, Hayes’ ‘PROCESS’ tool (model 4) was used with 10.000 bootstrap re-samples [35]. Group allocation was the independent variable, insomnia severity the dependent variable, and DBAS and PSAS the mediator variables, in two separate models. We used the pre- to post-test change scores of the ISI to correct for baseline values. Mediation was tested using the 95% confidence interval of the indirect effect (*path ab*).

## Results

Participant’s demographic characteristics can be found in Table 2. The mean age of the sample was 22.8 (SD = 4.2) and 74.3% of the participants were female. There were no significant differences between the groups at baseline (all *p*’s > 0.05). Even though none of the differences were statistically significant, the intervention group had a higher proportion of female participants (82.6%), higher proportion of international students (65.2%), and higher proportion of students consuming alcohol (78.3%) compared to the control group. The study compliance was very high, as 94% of all students completed T1. Compliance with the sessions in the intervention group was also high, since almost all students in the intervention group (*n* = 21, 95.5%) attended all four sessions, and one student attended the first three sessions. Two students (5.7%, *N* = 35) dropped out of the study, one in each group (one due to the death of a family member and one due to unknown reasons).

**Table 2:** Demographic characteristics of the study sample (*n* = 35)

	Smile	Waitlist	
Characteristic	*n* = 23	*n* = 12	
Age, mean (SD), years	23.5 (4.9)	21.4 (1.4)	*t*(33) = -1.44, *p* = .06
Gender, n (%)			
Female	19 (82.6)	7 (58.3)	
Male	4 (17.4)	5 (41.7)	*Χ²* (1) = 2.43, *p* = .12
Nationality, n (%)			
Dutch	8 (34.8)	7 (58.3)	
Other	15 (65.2)	5 (41.7)	*Χ²* (1) = 1.79, *p* = .28
Relationship status, n (%)			
In a relationship	6 (26.1)	5 (41.7)	
Married or cohabitating	2 (8.7)	-	
Single	15 (65.2)	7 (58.3)	*Χ²* (2) = 1.71, *p* = .43
Medication, n (%)			
No medication	19 (82.6)	11 (91.7)	
Antidepressants	1 (4.3)	-	
Non-psychoactive medication	1 (4.3)	1 (8.3)	
Iron supplements	2 (8.7)	-	*Χ²* (3) = 1.86, *p* = .60
Alcohol use, n (%)			
Yes	18 (78.3)	7 (58.3)	*Χ²* (1) = 0.22, *p* = .26
No	5 (21.8)	5 (41.7)	
Insomnia Severity (ISI), mean (SD)	14.3 (3.1)	15.7 (5.4)	*t*(33) = 0.98, *p* = .12
Depressive symptoms (BDI II), mean (SD)	11.9 (7.1)	11.5 (6.0)	*t*(33) = -0.15, *p* = .67
Anxiety symptoms (HADS-A), mean (SD)	8.9 (3.3)	8.8 (4.7)	*t*(33) = -0.06, *p* = .50
Quality of Life (Q-LES-Q), mean (SD)	54.7 (13.6)	61.2 (10.1)	*t*(33) = 1.45, *p* = .37
Pre-Sleep Arousal (PSAS), mean (SD)	42.6 (8.8)	42.1 (10.6)	*t*(33) = -0.16, *p* = .41
Dysfunctional Beliefs and Attitudes about Sleep (DBAS), mean (SD)	73.2 (19.8)	78.4 (20.8)	*t*(33) = -0.47, *p* = .97

*Note*. Non-psychoactive medication: Ibuprofen *n* = 1; Antibiotics *n* = 1

### Treatment effects on insomnia severity

Repeated measures ANOVA was conducted to test whether the SMILE intervention was effective in reducing insomnia severity compared to a control group in an intention-to-treat analysis (*n* = 35). There was a significant time x treatment effect *F*(1, 33) = 5.91, *p* = .02, *η2* = 0.15, in the unadjusted model. The main effect of time was significant *F*(1, 33) = 18.46, *p* < .001, *η2* = 0.36. The main effect of group allocation was non-significant *F*(1, 33) = 3.31, *p* = .08, *η2* = 0.09. Sensitivity analysis with complete cases showed similar results, with a significant interaction effect of *F*(1, 31) = 5.74, *p* = .02, *η2* = 0.16. Those who received the SMILE intervention had significantly lower insomnia severity (M = 10.7, SD = 4.8) compared to the waitlist at post-test (M = 14.7, SD = 4.9), *t* (33) = 2.33; *p* = .03, [95% CI: 0.51 to 7.52], representing a large effect with Cohen’s *d* = 0.83, [95% CI: 0.01 to 1.55].

### Clinically significant improvement of insomnia

In the SMILE group, 47.8% of the students were treatment remitters with a post-test insomnia severity score of less than 10, compared to 16.7% in the waitlist group [*X*^*2*^ (1, *N* = 35) = 3.28, *p* = .07]. In the SMILE group, 9.4% of students (*n* = 3) were treatment responders with a reduction in total ISI score of ≥ 8 points, compared to no responders in the waitlist group[*X*^*2*^ (1, 35) = 1.5, *p* = .22].

### Treatment effects on secondary mental health outcomes

**Fig. 2:**
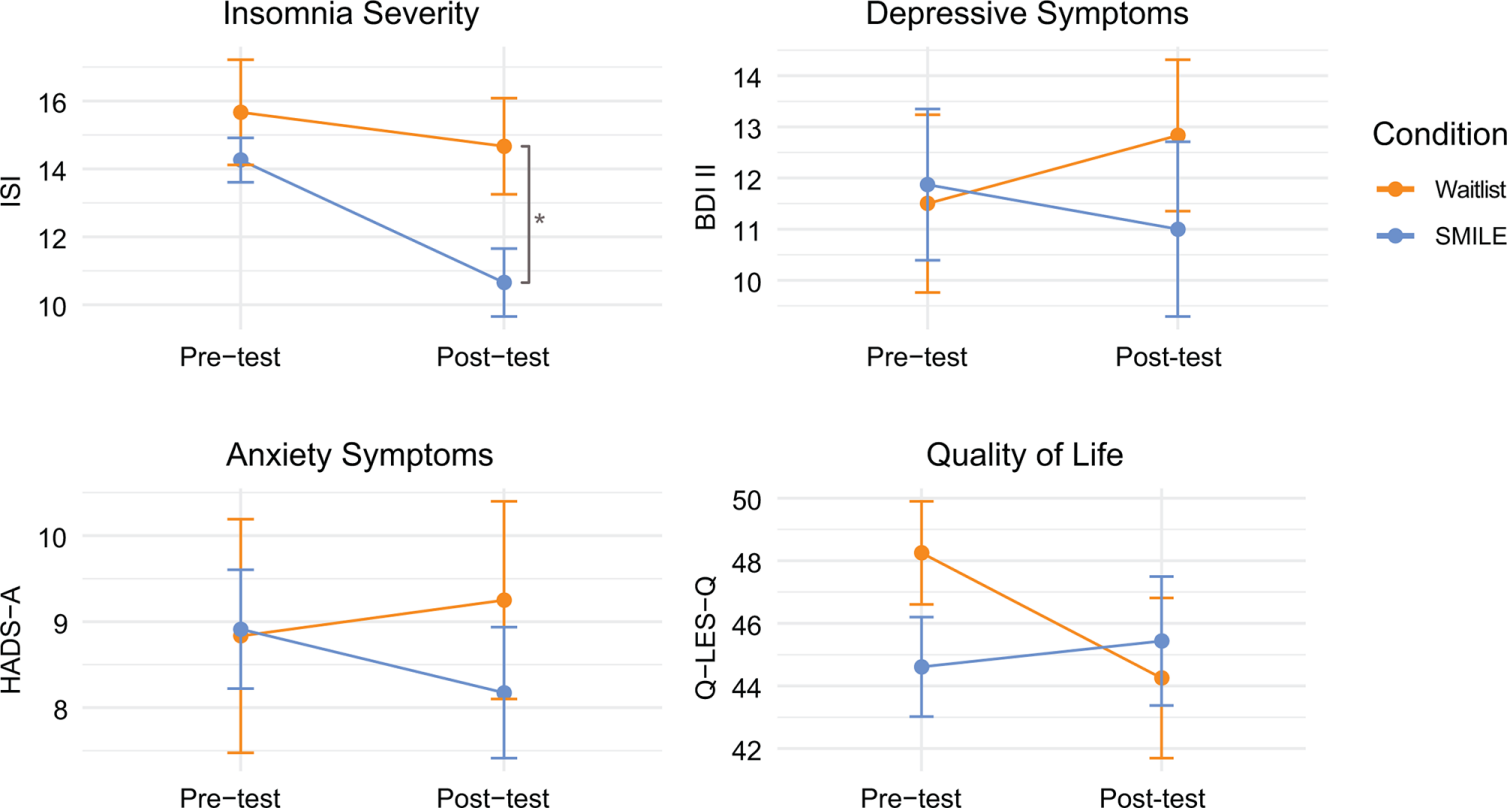
Interaction plots for pre- and post-test means for SMILE and waitlist. *Note* Means of Insomnia Severity (ISI), Depressive Symptoms (BDI-II), Anxiety Symptoms (HADS-A), and Quality of Life (Q-LES-Q) in SMILE and waitlist group at pre-test and post-test. Error bars represent standard error of the mean. * Significant mean difference *p* < .05

Results of repeated measures ANOVA indicated that for anxiety symptoms group x time interaction effects were non-significant *F*(1, 33) = 2.21, *p* = .15, *η2* = 0.06. Differences between SMILE and waitlist at post-test were non-significant as well with *t* (33) = 0.80, *p* = .43, and a Cohen’s *d* = 0.29, [95% CI: -0.42 to 0.99]. For depressive symptoms, repeated measures ANOVA showed that there was no significant group x time interaction effect for depression either, *F*(1, 33) = 1.82, *p* = .19, *η2* = 0.05. There were no significant differences between groups at post-test, *t* (33) = 7.03, *p* = .49 and Cohen’s *d* = 0.25, [95% CI: -0.45 to 0.95]. Results of repeated measures ANOVA on quality of life furthermore revealed no group x time interaction effect on quality of life, *F*(1, 33) = 2.29, *p* = .14, *η2* = 0.07. Post-test differences between the two groups were also not significant *t* (33) = -0.35, *p* = 0.73, with Cohen’s *d*= -0.12, [95% CI: -0.82 to 0.58]. Table 3 presents the results for primary and secondary outcomes. Figure 2 shows the interaction plots for pre- and post-test means of the main outcomes.

**Table 3:** Primary and secondary outcomes by treatment groups with intention-to-treat analysis

	SMILE (*n* = 23)			Waitlist (*n* = 12)							
	Pre-test	Post-test	Cohen’s d T0-T1	Pre-test	Post-test	Cohen’s d T0-T1	Time		Group x Time		
**Primary outcome**	Mean (SD)	Mean (SD)	Pre-Post	Mean (SD)	Mean (SD)	Pre-Post	F	*p*	F	*p*	*η2*
Insomnia Severity (ISI)	14.3 (3.1)	10.7 (4.8)	0.89	15.7 (5.4)	14.7 (4.9)	0.20	18.46	<0.001	5.91	0.02*	0.15
**Secondary outcomes**											
Anxiety symptoms (HADS-A)	8.9 (3.3)	8.2 (3.6)	0.20	8.8 (4.7)	9.3 (4.0)	0.11	0.17	0.68	2.21	0.15	0.06
Depressive symptoms (BDI II)	11.9 (7.1)	11.0 (8.2)	0.11	11.5 (6.0)	12.8 (5.1)	0.23	0.08	0.78	1.82	0.19	0.05
Quality of life (Q-LES-Q)	54.7 (12.8)	56.1 (17.6)	0.09	61.2 (10.2)	54.0 (15.8)	0.54	0.99	0.33	2.29	0.14	0.07
Pre-Sleep Arousal (PSAS)	42.5 (9.0)	35.7 (10.1)	0.71	43.5 (10.0)	43.1 (10.5)	0.04	3.76	0.06	3.76	0.06	0.09
Dysfunctional Beliefs and Attitudes about Sleep (DBAS)	81.8 (19.8)	67.9 (13.6)	0.82	78.4 (20.8)	79.8 (19.1)	0.07	4.05	0.05	6.20	0.02*	0.17

* *p* < 0.05

### Mediation analysis

The mediation analysis included group allocation as the independent variable and insomnia severity as the dependent variable. The pre-to-post change scores of DBAS and PSAS were included as mediator variables in two separate models. For intention-to-treat analysis, the total effect (*path c*) of group allocation on insomnia severity was significant with *b*= -2.61, [95% CI: -4.79 to -0.43].

As shown in Table 4, the effect of the SMILE intervention on insomnia severity was mediated by dysfunctional beliefs with *b* = 1.12, [95% CI: -2.58 to -0.06], meaning a decline in dysfunctional beliefs was related to a decline in insomnia severity. 54.7% of the variance was explained by the mediator DBAS. Pre-sleep arousal did not significantly mediate the effect of the SMILE intervention on insomnia severity with *b*= -0.68, [95% CI: -2.12 to 0.03]. 50.9% of the variance was explained by the mediator PSAS. Mediation analysis for completers showed the same pattern as for intention to treat.

**Table 4:** Dysfunctional beliefs and attitudes and pre-sleep arousal as mediators of the intervention effect on insomnia severity

M	DV	Effect of independent variable on mediator (a)	Effect of mediator on dependent variable (b)	Indirect effect (ab), [95% CI]	Direct effect (c’)	Total effect (c)
DBAS	ISI	b= 15.33, t= -2.51, SE= 6.10 *	b= 0.07, t= 2.59, SE= 0.28 *	b= -1.12, [-2.58; -0.06] *	b= -1.49, t= -1.40, SE = 0.65	b= -2.61, [-4.79 to -0.43] *
PSAS	ISI	b= -6.14, t= -1.77, SE= 3.47	b= 0.11, t= 2.15, SE= 0.05*	b= -0.68, [-2.12; 0.03]	b= -1.93, t= -1.81, SE= 1.07	b= -2.61, [-4.79 to -0.43] *

*Note.* M = Mediator variable, DV = Dependent variable, Independent variable = Group allocation, DBAS = Dysfunctional Beliefs and Attitudes about Sleep Scale, PSAS = Pre-Sleep Arousal Scale, ISI = Insomnia Severity Index. * *p* < .05

## Discussion

We found that the SMILE intervention, compared to the waitlist control group, significantly reduced insomnia severity. This finding is in line with previous research in university students, that has shown how the individual components of the SMILE intervention, such as CBT and mindfulness, are beneficial in improving sleep [13, 14, 36]. Recent meta-analyses showed that brief psychological interventions, especially CBT-I, had moderate to large effects in improving sleep outcomes in students [10, 13, 14], and mindfulness-based stress reduction had moderate effects on sleep quality in insomnia patients [23]. Also, the combination of both CBT and mindfulness as an integrated intervention in six weekly sessions was associated with improvements in sleep outcomes and a reduction in sleep-related arousal in adults with primary insomnia [37]. A recent systematic review concluded that multi-component interventions show moderate effects in improving university students’ sleep and mental health [17]. The effect size for insomnia severity in our study was *η2* = 0.15, which corresponds to *d* = 0.84 [95% CI: 0.63 to 1.14], and was larger than the moderate effect size of *d* = 0.55 reported by Chandler et al. [17]. This could be attributable to the enhancing effects of mindfulness combined with CBT-I [24] and also to the active discussion of student lifestyle struggles in the group sessions that the students seemed to benefit from. Another explanation could be the non-active comparison group. Chandler et al. found that studies with participants experiencing insomnia (versus those who did not) and those with a passive comparison group (waiting list or TAU) showed significantly higher effect sizes [17], which are two characteristics present in this study as well. Long-term effects of such interventions need to be examined in future studies; a meta-analysis on CBT for insomnia has shown that effects on insomnia severity and other sleep outcomes decline but remain clinically significant after one year [38].

Our results showed remission rates of approximately 50% (reaching below 10 on the ISI), while treatment response (reduction of 8 points) was much lower (approximating 10%). This is probably due to the baseline levels of insomnia being subthreshold to moderate in our sample. Therefore, the intervention seems to be effective in achieving remission in those with less severe insomnia, but it is unknown whether similar effects could be observed with a more severely symptomatic sample. Nevertheless, the multi-component part of the intervention is likely to have helped students, who are confronted with several challenges at this stage of their lives and might need a variety of tools to address them. Even though mediation analyses have shed light to some mechanisms, it remains difficult to disentangle which parts of this multi-component intervention were the most effective. Future studies could employ dismantling study designs to untangle effects of the different components in such interventions.

No significant effects were found on any of the secondary outcomes. Mental health symptoms either showed a slight positive trend of improvement or remained stable in the intervention group but generally slightly declined in the waitlist group, as shown in Fig. 2. That the improvement in mental health symptoms was not significant is probably a result of low statistical power, but further research needs to verify this in a larger sample. Still, effect sizes for depression, anxiety severity, and quality of life in our study were *d* = 0.02 [95% CI: -0.35 to 0.37], *d* = 0.15 [95% CI: -0.16 to 0.53], and *d* = 0.09 [95% CI: -0.25 to 0.42], respectively. Prior research has shown large improvements in depression and anxiety symptoms after mindfulness-based stress reduction in adults [23]. Recent systematic reviews and meta-analyses overall have shown that the effects of CBT-I are moderate to large on mood symptoms and moderate on quality of life [39, 40]. In university students, however, the pooled effects in a meta-analysis of single- and multi-component sleep interventions were smaller than in adults on the outcomes of anxiety with *SMD*= -0.23 and depression with *SMD*= -0.30 [17]. Similar to our findings, no improvements in anxiety symptoms were found after a multi-component mindfulness-based group sleep intervention in an adolescent population [24]. These findings show that such sleep interventions might have more specific effects - only on sleep outcomes - in younger populations.

The mediating effect of the cognitive and arousal processes was examined. Dysfunctional beliefs about sleep significantly mediated the effects of the intervention on insomnia severity. This finding is in line with most previous literature, supporting the evidence of dysfunctional beliefs as a mediator of insomnia symptom improvement following CBT-I [41–45]. However, the current study design warrants cautious interpretation of the mediation results since we only established a co-occurring change of dysfunctional beliefs and change of insomnia severity. Therefore, although the finding is plausible we emphasize the need for more rigorous mediation research using multiple time points to elucidate the causal mechanisms underlying these associations. Dysfunctional beliefs are an important factor in the treatment of insomnia since it plays a role in increased anxiety around sleep and engagement in sleep-disrupting compensatory behaviors [25]. Future research might look into the specific beliefs that may change during insomnia treatment, for instance by means of Network Intervention Analysis [46]. In contrast, pre-sleep arousal did not significantly mediate the effects of the intervention on insomnia severity. In a previous systematic review and meta-analysis, five studies were summarized which included hyperarousal outcomes and they concluded that only limited evidence was found for hyperarousal as a mediator for CBT-I [44]. It remains challenging to establish causal links between treatment, mechanisms, and outcomes, especially in multi-component interventions such as the SMILE intervention. Still, future studies should consider a range of cognitive factors (e.g., sleep self-efficacy, locus of control) and behavioral factors (e.g., variability in sleep-wake time), as suggested by Schwartz and Carney [25], and should utilize rigorous designs with multiple measurements, in order to get a better understanding of how CBT-I works.

The high adherence to the intervention sessions highlights its feasibility and acceptability, though challenges in recruitment suggest the need for exploring alternative approaches. Adherence to the sessions was high (95.5% attending all sessions), although this is partly attributable to the flexibility of the group leaders, who provided a brief substitute session in case a participant was unable to attend the group session. Still, it is a good indicator of the acceptability of the intervention and feasibility of the current intervention design. The short duration makes this type of multi-component intervention easy to implement in a student population. On the other hand, the recruitment period was long and the recruitment rate was low, possibly indicating that not many students were willing or able to engage in the intervention. Therefore, it would be worthwhile to investigate alternative ways to improve sleep in students. A more accessible and scalable alternative to face-to-face group therapy might be internet-delivered interventions.

This study had several limitations and strengths. The most important limitation is the small sample size leading to decreased statistical power. The recruitment stop was due to the COVID-19 pandemic, yet again the recruitment rate was fairly slow so it is difficult to estimate how long it would have taken to complete the study. Furthermore, as mentioned before, adding a midpoint measurement would have given more information about temporal precedence in the mediation analysis. A final limitation is that the results may not be generalizable to other populations since our sample included a relatively heterogenous group of mostly female students. Nonetheless, the study has some strengths. The study design is a randomized controlled trial and analyses were done with the intention to treat-approach. Furthermore, as mentioned before, study drop-out was very low since only two participants (5.7%) dropped out.

## Conclusions

In conclusion, this study has shown that students with insomnia can benefit from a four-week group intervention in improving their insomnia symptoms. Multi-component interventions tailored to the needs of university students offer a promising path in improving sleep problems in this population at risk for both sleep and mental health disturbances. Still, the low number of treatment responders and low effect size in secondary outcomes point towards further investigation. The current treatment might be further examined in a series of case studies in order to deduce what elements need to be added or expanded to make the treatment more effective. To summarize, future research should include larger samples, multiple measurement moments to formally test mediation, and explore different forms of delivery to further examine the effectiveness of multi-component sleep interventions.

## Data Availability

Data will be deposited in DataVerse under the following link: https://doi.org/10.34894/0GSC6R.

## Abbreviations

ANOVA: Analysis of Variance
BDI: Beck Depression Inventory
CBT-I: Cognitive Behavioral Therapy for Insomnia
DBAS: Dysfunctional Beliefs and Attitudes about Sleep
ISI: Insomnia Severity Index
HADS-A: Hospital Anxiety and Depression Scale - Subscale Anxiety
RCT: Randomized Controlled Trial
PSAS: Pre-Sleep Arousal Scale
QoL: Quality of Life
Q-LES-Q: Quality of Life Enjoyment and Satisfaction Questionnaire
